# Practice Effects and Long Delays: A Case Report Exploring a Novel Approach to Detecting Accelerated Long-Term Forgetting

**DOI:** 10.1093/arclin/acaf077

**Published:** 2025-08-28

**Authors:** Chris Gaskell, Cleo Keeling-Ball, Callum Furniss, Jonathan Evans

**Affiliations:** North Staffordshire Combined Healthcare NHS Trust, Stoke, UK; Clinical and Applied Psychology Unit, University of Sheffield, UK; North Staffordshire Combined Healthcare NHS Trust, Stoke, UK; North Staffordshire Combined Healthcare NHS Trust, Stoke, UK; School of Health and Wellbeing, College of Medical, Veterinary and Life Sciences, University of Glasgow, UK

**Keywords:** Accelerated long-term forgetting, Temporal lobe epilepsy, Serial assessment, Practice effects

## Abstract

**Objective:**

Accelerated Long-Term Forgetting (ALF) is when newly learned information “decays” faster than expected over an extended period and is associated with temporal-lobe epilepsy (TLE). There is no well-established method for assessing ALF despite its apparent prevalence. We hypothesized that evidencing an absence of practice effects may represent an effective approach to detecting ALF. We sought to determine if this method, along with the long-delay memory tests, could evidence ALF in a single case.

**Method:**

We present a 66-year-old male with TLE who had memory complaints despite a stable memory profile over 4 years. Memory tests that employ a short (20–30 min) and a long delay (4 days) condition were used to assess forgetting, whereas repeatedly administered tests were used to detect practice effects. We anticipated poorer memory performance on the long versus short-delay test condition and a lack of improvement on memory tests that were repeated.

**Results:**

For repeat administration tests, there was a marked score increase, indicating practice effects, for verbal and visual domains. For long delay tests, however, there was a notable drop with retention falling in the exceptionally low range.

**Conclusions:**

These findings suggest a dissociation between long delay and serial assessment tasks for detecting ALF.

## INTRODUCTION

Accelerated long-term forgetting (ALF) is a process whereby an individual is able to learn and adequately recall new information over time periods typically used in standardized tests of memory (e.g., 30 mins) but shows an abnormally accelerated loss of this information from memory over subsequent days or weeks (Butler et al., 2019). ALF has most commonly been associated with temporal lobe epilepsy (TLE) and transient epileptic amnesia (TEA), however, it has more recently been explored in a range of other neurological conditions (e.g., Rodini et al., 2022).

Objective identification of ALF with cognitive assessment has been a methodologically challenging endeavor. Traditional neuropsychological memory tests do not typically assess recall beyond one testing session, with delay periods rarely exceeding 30 min. It is proposed ALF may not be readily detectable using such time lengths as the divergence between ALF and healthy memory trajectories have not yet occurred or become significantly different (Mameniškienė et al., 2020). The extent to which ALF represents (a) a continual steady decline that eventually becomes detectable; versus (b) initial stability before later showing an abrupt divergence (drop in performance), remains open to debate and casts some uncertainty upon the naming of ALF. This therefore raises the question of whether we can be sure there is an acceleration (A) and does this truly represent “long term” forgetting (LF) (as opposed to continual/gradual forgetting).

It has been argued that the identification of earlier divergence in memory performance between ALF and healthy controls has been confounded by a series of methodological challenges (Elliot et al., 2014). In a critical review of the literature, Elliot and colleagues highlighted a series of considerations for robust assessment, including ceiling/floor effects, patient rehearsal, control matching issues, and selection of long retest intervals (e.g., second delayed recall at 1 week). We would argue that additional important considerations include: the reliance upon single trials (usually from list learning tasks), which often have poor test–retest reliability, and small (if any) accompanying normative data. We also think further consideration is required as to how assessment of ALF can feasibly be routinely adopted by clinicians using the collection of tests that are already available, as opposed to the perhaps unnecessary need to develop new tests. With that in mind, this paper focuses on the novel combination of two distinct and existing approaches to assessment: (1) extended delay periods, and (2) detection of “practice effects” across repeated administrations.

### Practice Effects

Practice effects occur when there is an improvement in an individual’s cognitive test score across two or more time points that is not directly attributable to the underlying cognitive skill being sampled (Calamia et al., 2012). This phenomenon occurs across most (if not all) cognitive tests, with meta-analytic evidence estimating an overall effect-size of almost 0.25 of a standard deviation across studies analyzed (Calamia et al., 2012). This improvement is thought to be due to increased familiarity with test content, but also due to learning how to approach the test and the development of an effective strategy (Hinton-Bayre & Kwapil, 2017). The latter issue likely explains why practice effects are not eliminated when participants complete alternative forms (i.e., the same test format with different content/items) subsequent to completion with the standard test form (e.g., Beglinger et al., 2005). As practice effects are likely to reflect underlying learning processes, it is perhaps unsurprising that improvements on memory tests across time points can be particularly large. For example, individuals who complete the California Verbal Learning Test – second edition (CVLT-II) are reported to recall an additional 8 words on average across five learning trials when retested a mean of 21 days later (Delis et al., 2000). The magnitude of the practice effect can vary for different reasons with research showing that practice effects are reduced with increasing time intervals between test administrations (Calamia et al., 2012). Individuals with a higher IQ may also be more likely to benefit from practice on cognitive tests (Rapport et al., 1997).

Although many consider practice effects to be a “nuisance” and something to be accounted for when assessing change between administrations (e.g., through standardized regression-based change models [SRBs]), some researchers have re-considered their potential clinical applications (e.g., Duff et al., 2011). For example, reduced, or an absence of, practice effects over a short period has been found to be a reliable risk marker for the diagnosis of dementia or risk of future decline in cognition (Jutten et al., 2020). As practice effects are likely to be reliant on efficient encoding and consolidation of information over the longer term (e.g., a week between administrations), individuals with ALF in TLE may be less likely to benefit from repeated administration of tests over such time intervals. An absence of such practice effects on memory tests may, therefore, present an alternative approach to detecting ALF in TLE in clinical practice. To the authors’ knowledge, however, the specific measurement of practice effects within this clinical context has not been explored.

### Aims

The current case report explores the extent to which a single patient with TLE and suspected ALF demonstrates cognitive performance in line with clinical hypotheses based on assessment using very long delays and serial administration (i.e., detection of practice effects). The clinical hypotheses were that: (1) performance for memory tests would be preserved over traditional delay periods (20–30 min); (2) serial assessment over a fixed interval (4 days) would not result in demonstrable practice effects; and (3) for memory tests with both a short (20–30 min) and long delay (i.e., several days), the patients performance would be better on the former vs. latter.

## CASE PRESENTATION

A 66-year-old, right-handed male (here called CM – pseudonymised initials) attended for neuropsychological assessment in 2024. CM had experienced memory difficulties dating back to 2020, which had previously been assessed through three separate neuropsychological evaluations. Memory concerns arose several months following the diagnosis of TLE. TLE had a sudden onset, and no underlying etiology was identified despite investigation. At the time of the assessment, the cause of TLE remained unknown.

The nature of the memory concerns, as reported by CM’s wife and daughter, related to episodic memory. This included memory for recent (e.g., difficulty recalling what happened the previous day) and more distant events (e.g., he was unable to recall anything from his daughter’s wedding). CM had also been observed as showing difficulty recalling familiar routes and with prospective remembering (e.g., appointments, and anniversaries). In earlier neuropsychology evaluations, CM had denied his family’s concerns regarding his memory, although when sensitively provided with evidence of difficulty, he would adjust his view accordingly (until later forgetting).

During the current evaluation (2024), CM and his family reported that there had been clinical improvement since the recent cessation of seizures but that some difficulty with memory persisted. CM was reported to still require regular prompting and supervision. CM considered the mild improvements to be a result of gaining seizure control and optimizing his lifestyle (e.g., eating healthy, and improved sleep).

### Prior Input

Due to the combination of epilepsy emerging in later life and concerns from his family that his memory difficulties were progressive it was initially queried whether CM has an underlying neurodegenerative condition. This led to serial neuropsychological assessments (2020, 2022, and 2023), multiple neuroimaging studies, and a specialist assessment by a tertiary care rare dementia service. Each of the neuropsychological assessments (as detailed in the results section) could not substantiate the memory concerns reported by CM or his family; in fact, cognitive test performance indicated that CM’s memory and broader cognitive functioning were strong and stable over 4 years. This is despite CM reporting, at follow-up, that he had no memory of the clinician, the building, or the experience of undergoing cognitive testing. CM could, however, recognize the clinician and recall previous conversations between appointments within a single testing period (i.e., 1–2 weeks apart).

### Health

CM’s health history included well-controlled hypertension, and he had never previously accessed support for mental health. There was no family history of epilepsy or neurodegenerative disease. In terms of seizure management, CM was initially trialed on Phenytoin and later Levetiracetam, however, there was no therapeutic benefit shown. He was later prescribed Lamotrigine (December 2022), which demonstrated a strong therapeutic benefit, leading to seizure remission. Since February 2023. He had previously experienced seizures twice per month.

MRI scans of the patient’s brain suggested mild non-progressive atrophy in bifrontal and parietal areas. An FDG-PET brain scan in December 2021 was reported as not being suggestive of a neurodegenerative condition. The cause of the fronto-parietal atrophy was unclear, and the neuroradiology report recommended correlation with clinical findings. In this context, the initial neuropsychological assessment was prompted by suspicion of a progressive neurodegenerative condition, given the presence of atrophy and seizure onset. However, this was subsequently ruled out based on longitudinal monitoring of clinical and neuropsychological findings, as outlined in the *Results* section.

## METHODS

### Materials and Procedure

During the current neuropsychological evaluation (2024) CM maintained concerns about his memory despite a history of strong and stable performance on traditional neuropsychology memory tests. At this stage, CM was queried regarding further atypical memory problems and provided informed consent for an extended period of testing to enhance understanding of his memory difficulties. He was informed of the experimental approach of the assessment but was not made aware of the specific test procedures or clinical hypotheses to avoid influencing the clinical assessment. Upon completion of the assessment, CM received feedback on his neuropsychological test results and was provided with supportive cognitive rehabilitation. Additionally, he reviewed the current manuscript and actively contributed to its content (see Acknowledgments section).

The additional testing period allowed for the assessment of memory consolidation over an extended period using two different methods, long delay recall and serial administration. Long delay recall was assessed using the BIRT Memory and Information Processing Battery – Second Edition (BMIPB-II; Oddy et al., 2019), and serial administration included the California Verbal Learning Test – Third Edition (CVLT-III; Delis et al., 2017) and the Rey Complex Figure Test (RCFT, Meyers & Meyers, 1995).

The BMIPB-II test battery assesses a broad range of memory and information processing abilities. For the current assessment, only the verbal memory subtests were administered, selected for their inclusion of a long-delay recall interval (7–10 days post-exposure), in addition to a standard delay (30–40 min). These subtests include a list learning task and a story memory task. The CVLT-III involves a 16-word list learning test, incorporating immediate recall (following interference) and delayed recall intervals (20 min). The RCFT is a visual memory assessment where participants are asked to reproduce a complex geometric figure both immediately (after 3 min) and after a delayed interval (30 min) following an initial copy condition.

The administration of standard delay testing (BMIPB-II, CVLT-III, RCFT) took place in the first appointment and then long delay recall (BMIPB-II) and re-administration (CVLT-III and RCFT) in the second appointment, 4 days later. The long delay recall was to establish if memory performance became abnormal over an extended period, whereas serial administration was to establish if practice effects took place. The presence of practice effects would theoretically be supportive of memory consolidation. The use of a 4-day delay deviated from the procedures prescribed in the BMIPB-II (7–10 days); this was due to difficulty matching the availability of the clinician and patient. It was acknowledged before administration that this shorter delay might increase the likelihood of the patient performing well, potentially reducing the sensitivity of the assessment. When commencing this evaluation, CM did, for the first session, remember the clinician from the previous assessment.

The historic neuropsychological assessment data (2020, 2022, and 2023) is provided as evidence of memory proficiency as far as can be tested with traditional assessments using a delay of 20–30 min. This included repeat administrations of the Wechsler Adult Intelligence Scale, Fourth Edition (WAIS IV; Wechsler, 2008) and the Wechsler Adult Memory Scale, Fourth Edition (WMS IV; Wechsler, 2009) with reference to an estimate of premorbid functioning as assessed using the Test of Premorbid Functioning (ToPF). These test data allowed for the assessment of cognitive functioning and durability of intelligence and memory abilities. In short, the WAIS-IV assesses intellectual functioning, with sub-domains including verbal comprehension, perceptual reasoning, working memory, and processing speed. The WMS-IV allows for assessment of learning, immediate memory recall, and short-term consolidation (20–30 min) for verbal and visual memory, in addition to assessment of visual working memory.

CM’s strengths and weaknesses on the WAIS-IV were assessed by comparison to the mean composite score with point estimates of abnormality calculated using the approach described by Crawford and colleagues (2011).

WAIS-IV and WMS-IV index scores were also assessed for statistically significant change over time using standardized regression-based models using the approach offered by Crawford and Garthwaite (2006). This method estimates the patient’s score using the previous test score while adjusting for regression to the mean, practice effects, and measurement error associated with the cognitive tests employed. There is a lack of normative data for retest performance for these tests using the time intervals used in the current administration of the WAIS-IV and WMS-IV (˃12 months). Consequently, the normative data from the test manual (mean re-test period of 22 days) was used whereas acknowledging that practice effects are likely to be reduced here due to longer retest intervals.

### Current Assessment

First, memory tests inclusive of a long-delay condition were employed to provide a norm-referenced estimate of memory at 4 days post-exposure to the stimuli. Consistent with the BMIPB-II procedure, the patient was not aware that the follow-up consultation would be to assess memory, and he was not informed of any need to recall information from the first testing session. This included the story memory and list learning subtests from the BMIPB II. BMIPB-II raw scores were converted to standard scores using the electronic scoring assistant accompanying the test manual, which applied a continuous norming approach with age and years of education as predictor variables. For BMIPB-II subtests not available within the regression-based scorer, standard scores and cumulative percentages are provided from the test manual, as shown in Table 2.

Second, two commonly used memory tests for which CM had no prior experience were employed with repeat administration (4 days apart). This was to test the clinical hypothesis that long-term forgetting would be associated with an absence of practice effects. For this, the CVLT-III and the RCFT were employed. Alternative forms were not employed to enhance the opportunity for CM to demonstrate practice effects. The CVLT-III included continuous regression-based normative data (Delis et al., 2017) using the Q-interactive™ platform, whereas for the RCFT, normative data from the user manual (Meyers & Meyers, 1995) was employed.

Assessments were presented in sequence, with the CVLT administered first, followed by the RCFT, and finally the BMIPB-II. The size of the discrepancy between scores (e.g., BMIPB-II standard delay and long delay) was expressed as rate of abnormality (i.e., base rate) using the T distribution approach offered by Crawford and Garthwaite (2002).

In line with the clinical hypotheses outlined in the introduction, it was expected that:

1. Tests with traditional delay intervals of 20–30 min (CVLT-III, RCFT, and standard delay subtests of the BMIPB-II) would be in the average range (or above).
2. Tests with traditional delay intervals of 20–30 min (CVLT-III, RCFT, and standard delay subtests of the BMIPB-II) would not show pronounced practice effects across repeat administrations.
3. Subtests with long delay intervals (long delay subtests from the BMIPB-II) would show scores in the impaired range (determined as 2 SD below average) or a notable reduction from performance in the standard delay conditions.

## RESULTS

### Historic Assessments

The results of the preceding neuropsychological assessments are shown in Table 1 (an illustration is available in the Supplementary Materials; see Supplementary material online, *Figure S1*).

**Table 1:** Historical Neuropsychological Test data. Cognitive test scores for the WAIS-IV and WMS-IV from previous neuropsychological assessments conducted in 2020 (T1), 2022 (T2), and 2023 (T3)

	Time 1 (2020)	Time 2 (2022)	Time 3 (2023)	ToPF
**Intelligence (WAIS IV)**				
Verbal Comprehension	108 (102–113)	114 (108–119)	120 (114–125)^a^	109
Perceptual Reasoning	127 (119–132)	133 (125–138)	133 (125–138)	110
Working Memory	—	122 (114–127)	128 (120–133)	112
Processing Speed	—	97 (89–106)	111 (102–118)	103
Full Scale	—	123 (117–126)	130 (125–133)	110
**Memory (WMS IV)**				
Auditory Memory	105 (99–111)	112 (105–118)	102 (96–108)^b^	—
Visual Memory	108 (102–113)	103 (97–109)	117 (111–122)	—
Visual Working Memory	106 (98–113)	115 (107–121)	123 (114–129)	106
Immediate Memory	123 (116–128)	113 (106–119)	111 (104–117)^c^	104
Delayed Memory	94 (88–101)	104 (97–111)	110 (103–116)	104

^a^Significant Improvement (T1 vs. T3).

^b^Significant Reduction (T2 vs. T3).

^c^Significant Reduction (T1 vs. T3).

*Note:* ToPF = Test of Pre-Morbid Functioning; WAIS IV = Wechsler Adult Intelligence Scale, Fourth Edition; WMS IV = Wechsler Adult Memory Scale, Fourth Edition.

### Intelligence

The first administration of the WAIS-IV (T1), which included only two domains, found perceptual reasoning performance to fall above premorbid estimates (index score = +16.6), a score discrepancy of this magnitude would be expected to occur in 9.14% of the population (i.e., base rate). Verbal comprehension was as estimated (index score = −0.8; base rate = 46.18%). In terms of strengths and weaknesses, the first fully administered WAIS-IV (T2) identified perceptual reasoning ability as a statistically significant strength (*p* = <0.05, base rate = 2.12%) and processing speed as a statistically significant weakness (*p* = <0.05, base rate = 2.36%). The Mahalanobis distance index indicated that 9.53% of the normative population would be expected to show a more unusual overall profile.

There was a trend for scores to improve over time however this was only significant for verbal comprehension between T1 and T3 (Z = +1.96). Each of the other intellectual domains, including the full-scale IQ score, demonstrated an absence of statistically significant change across assessments.

### Memory

The first administration of the WMS-IV (T1) indicated that CM had outperformed his premorbid estimate for immediate memory (index discrepancy = +19.1, base rate = 5.84%) whereas delayed memory fell below estimates (index discrepancy = −9.8, base rate = 21.69%), and visual working memory performance was consistent with premorbid estimates (index discrepancy = −0.2, base rate = 49.20%).

In terms of forgetting scores (immediate memory vs. delayed memory), this was statistically significant for T1 (index discrepancy = 29, base rate = <0.00%) but not for T2 or T3.

When looking at the change in WMS-IV score over T1 vs. T2 vs. T3, performance was generally stable, with the exceptions of a statistically significant reduction in scores for immediate memory (T1 vs. T3, z = −2.68), and auditory memory (T2 vs. T3, z = −2.30). It is worth noting that the negative Z scores are partly influenced by adjustments for practice effects, which are harder to achieve for individuals with baseline scores at the upper end of the normal distribution.

### Current Evaluation

The results of the current neuropsychological evaluation assessing long-term forgetting are shown in Table 2.

**Table 2:** Cognitive test scores for tests performed in the serial assessment. Note the intervals is 4 days apart

	**Time 1**		**Time 2**		**Change**
	Raw	Standardized	Raw	Standardized	
**California Verbal Learning Test (CVLT 3)**					
**Subtests Scores:**					
Immediate I	4	T = 45	10	T = 70	+25
Immediate II	9	T = 57	12	T = 68	+11
Immediate III	10	T = 52	16	T = 79	+27
Immediate IV	10	T = 52	13	T = 61	+9
Immediate V	11	T = 52	12	T = 55	+3
Immediate B	3	T = 41	6	T = 61	+20
Short Delay Free	12	T = 62	15	T = 75	+13
Short Delay Cued	10	T = 49	14	T = 65	+16
Long Delay Free	12	T = 58	16	T = 74	+16
Long Delay Cued	11	T = 52	14	T = 62	+10
Recognition Hits	12	T = 36	16	T = 68	+32
Recognition False +	0	T = 66	2	T = 51	−15
Recognition Discrimination	92	T = 51	96	T = 56	+5
**Composite Scores:**					
Trials 1 to 5	50	T = 52	72	T = 70	+18
Delayed Recall	44	T = 56	59	T = 69	+13
Total Recall	101	T = 52	144	T = 71	+19
**Rey Complex Figure Test (RCFT)**					
Immediate Recall	25.5	T = 74	33	T = 80	+6
Delayed Recall	22	T = 66	33	T = 80	+14
Recognition Total	21	T = 54	23	T = 67	+13
Copy	36	>16%^ile^	36	>16%^ile^	—
Time to Copy	79 s	>16%^ile^	65 s	>16%^ile^	—
					
**BIRT Memory & Information Processing Battery (BMIPB II)**					
**Story Memory:**					
Immediate Recall	25	T = 45	—	—	—
Delayed Recall	34	T = 58	—	—	—
Long Delay Recall	—	—	6	2–5%^ile^	—
Long Delay Total	—	—	14	2–5%^ile^	—
Long Delay Retained	—	—	18%	<2%^ile^	—
**List Learning:**					
Trials A1 to A5	44	T = 43	—	—	—
Trial A5	13	50%^ile^	—	—	—
Trial B	6	50%^ile^	—	—	—
Trial A6	11	50%^ile^	—	—	—
Intrusions	1	25–95%^ile^	—	—	—
Recognition A Words	15	10–95%^ile^	—	—	—
Recognition B Words	13	50%^ile^	—	—	—
Recognition Total	28	50%^ile^	—	—	—
Recognition List A	13	25%^ile^	—	—	—
Recognition List B	13	25–50%^ile^	—	—	—
Recognition Total	26	25–50%^ile^	—	—	—
Long Delay List A Words	—	—	0	2–10%^ile^	—
Long Delay List B Words	—	—	0	2–50%^ile^	—
Long Delay List Correct Recall	—	—	0	2–10%^ile^	—
Long Delay List Identification	—	—	0	2–10%^ile^	—

#### Learning and immediate recall

CM’s performance for learning was in the average range based on the CVLT-III learning trial composite ($T$ = 52) and the BMIPB list learning trials ($T$ = 43). For immediate recall, CM scored in the high average range for the CVLT-III ($T$ = 62), the average range for BMIPB story memory ($T$ = 45), and the average range for BMIPB post-interference (50th percentile). For visual memory, CM performed within the above-average range ($T$ = 74) for immediate recall of the complex geometrical figure from the RCFT.

#### Delayed recall

For standard delay memory tests (20–30 min), CM showed comparable (non-significant) performance ($T$ = 56, discrepancy = +4) to immediate memory of verbal lists from the CVLT-III. For story memory of the BMIPB-II, there was a non-significant improvement for delayed recall ($T$ = 58, discrepancy [[ineq08]]= +13) compared to immediate recall. For delayed visual memory (RCFT), there was a non-significant reduction in score ($T$ = 66, discrepancy = −8).

#### Serial administration

Serial administration included the CVLT-III and RCFT administered repeatedly, with a 4-day interval.

For verbal memory (CVLT-III) CM made improvements of between 1 and 2 SD for composite scores on the second administration, including learning trials ($T$ = 70, discrepancy = +18), delayed recall ($T$ = 69, discrepancy = +13), and total recall ($T$ = 71, discrepancy = +19). Similarly for visual memory (RCFT), comparable scores were shown for primary outcomes of immediate recall ($T$ = 80, discrepancy = +6), delayed recall ($T$ = 80, discrepancy = +14), and recognition ($T$ = 67, discrepancy = +13).

#### Long delay recall

For the long delay (BMIPB-II), testing included story memory (free recall, total recall, retention), and list recall. For story memory, CM scored in the below-average range for long delay free recall (z = −1.56). This contrasted in performance to the standard delay recall, with a discrepancy size that would be expected to occur (i.e., base rate) in 6.25% of the population. For total recall, performance was in the below-average range (z = −1.74, base rate = 6.25%), and finally, for retention, CM was in the exceptionally low range (z = −2.33, base rate = 1.18%). For the list recall long delay conditions, CM was unable to recall any words.

## DISCUSSION

This case report is the first known attempt to use practice effects through standardized regression-based models as a means of detecting ALF associated with TLE. Using serial assessment of common memory tests 4 days apart, we observed strong practice effects which were not expected due to the suspected role of long-term forgetting. To our surprise, this was conflicting with data retrieved from the long delay subtests which indicated impairment for memory consolidation/retention. These findings are suggestive of dissociation in these memory tasks, with long-delay tests showing as more sensitive to the effects of long-term forgetting than the absence of practice effects.

CM demonstrated comparable performance across immediate and shorter delayed conditions on the BMIPB-II, CVLT-III, and RCFT. The presence of practice effects—though unexpected—raises questions about the role of repeated exposure in reactivating weak memory traces. Further support for this has been demonstrated by other researchers of ALF. For example, Jansari and colleagues (2010) found that repeated recollection improves recall over time, as shown in story memory tasks assessed after 4 weeks. McGibbon and Jansari (2013) extended these findings using unfamiliar word pairs, showing that repeated recall maintained performance within normal limits for at least 24 h. Similarly, Ricci and colleagues (2019) demonstrated that encoding through repeated recall strengthens retention, sustaining normal performance for up to a week. These findings align with CM’s demonstrable practice effects based on the serial assessment 4-days apart.

These findings provide some evidence that CM’s difficulty with memory may primarily lie in episodic recall, with implicit memory being less affected. The practice effects observed indicate some consolidation of the test material, perhaps supported by implicit memory and recognition processes that support learning/re-learning. This supports the idea that additional recall opportunities at extended intervals could help sustain retention and consolidation in ALF and therefore may be a core aspect of effective cognitive rehabilitation interventions.

### Implications

In terms of clinical implications, this case report suggests that memory tests incorporating very long delays (e.g., 4 days) between acquisition and retrieval may be more sensitive to detecting accelerated long-term forgetting than traditional tests using a 30-min delay. Furthermore, the reliance upon conventional memory tests that do not assess memory retention beyond 20–30 min (after the initial recall period) risks impairments going undetected for those who have this form of memory difficulty. This is particularly relevant for the many people presenting for neuropsychological evaluation with TLE for which ALF has been commonly linked. To address this gap, researchers have developed their own materials and procedures (e.g., Laverick et al., 2021). Although some have extended the delay period in standardized tests, others have designed entirely new testing materials. Helmstaedter and colleagues (1998) attempted to assess ALF by examining participants’ recall of their testing session after a 1-week delay. However, their approach was problematic because there was no evidence to confirm that participants could recall the information on the day of the initial testing. This lack of baseline recall data makes it difficult to accurately infer forgetting rates and, consequently, ALF. Similarly, Isaac and Mayes (1999) utilized a multiple-presentation procedure for a story instead of the recommended method of extended exposure. These adaptations, while innovative, highlight the need for more refined tests that are sensitive to ALF and incorporate longer delays, thereby eliminating the necessity for researchers to create their own materials or procedures (Elliott et al., 2014). Long delay memory tests, such as the BMIPB-II, may be suited to the detection of long-term forgetting and clinicians may well already have access to the right tools. Despite the fact that the BMIPB-II has been available for several years now, with a large normative dataset for long delay conditions, we are not aware of any published reports of its use in the assessment of accelerated long-term forgetting.

As noted, the results suggest that memory consolidation and retrieval may be supported by re-activating the “weak trace” memory. In terms of supporting people with ALF, further investigations are needed to examine the therapeutic effect of rehearsal and cueing for overcoming the episodic memory difficulty. Preliminary evidence has emerged showing that rehearsal may help overcome consolidation challenges via aiding information storage in the long-term memory for people with TLE (Jansari et al., 2010).

Finally, this case report found evidence for durable difficulties in consolidation, beyond the point of seizure cessation. This highlights that seizure cessation alone may not address the memory deficits associated with ALF, and further considerations aimed at improving memory consolidation might be necessary to enhance long-term outcomes.

### Strengths

This case report provides the first comparison of long-term delay recall trials and practice effects in the assessment of ALF. It highlights long-delay memory tests, such as the BMIPB-II, as useful tools for detecting ALF. Although the serial assessment did not prove to be sensitive, this initial exploration offers valuable insights that advance our understanding of ALF. Further strengths of this case report include (1) availability of long-term comprehensive prior test data for a single patient; (2) application of a tool (BMIPB-II) which is inclusive of long delay trials and a large normative dataset; and (3) application of tools readily available to the local clinician for assessing ALF, avoiding the need for reliance upon experimental tests that are scarcely available to clinicians.

### Limitations

One limitation is that there may have been interference between tests administered within the same testing period. The BMIPB-II (list learning) and CVLT-III are both list learning and recall tests. Given these parallels, it becomes appropriate to think about both proactive and retroactive interference between the tests. During testing, the CVLT-III was administered before the BMIPB-II. The capacity to recall words from the BMIPB-II list may have been hampered by words learnt in the CVLT-III, when proactive interference is considered. Both proactive and retroactive interference can have complications for true memory performance in neuropsychological testing and for this reason, it is appropriate to consider the timing and sequence of tests used to minimize interference. Attempts were made to counteract interference effects with CM; between the administration of the BMIPB-II and the CVLT-III was a different natured test (RCFT) and a break. Future replications may also wish to consider using word lists that differ either thematically or contextually to help those undergoing testing to better segregate information.

Another important consideration is that testing was completed over 4 days. Elliott and colleagues (2014) state that similar research has often utilized delays of 1 week or less, but evidence of delays of up to 3 weeks has also been reported (Muhlert et al., 2011). Longer delays may reveal an accelerated effect on memory decay and can help detect subtle deficits in long-term memory consolidation and retrieval that are not as notable within a shorter time frame. Considering this, this case report is unable to make any contribution to the debate of forgetting being truly “accelerated” or not. It is also important to consider that establishing robust patterns of memory decay over extended periods can help to distinguish between normal forgetting and forgetting associated with conditions such as epilepsy or Alzheimer’s disease. As a result, it’s critical to consider that the delay period in this case report may have reduced the sensitivity and diagnostic value of the tests used for detection of ALF. Even so, one could argue that utilizing a shorter retest period while measuring impairment on long delay tasks yields higher confidence; an extended delay may only attenuate these findings, especially when querying how sensitive tests may be to the decay of information over longer periods of time. This point would also be helpful to consider when emphasizing that, although the 4-day delay did not align with the test manuals, it was fitting for the purpose of this case.

Finally, these findings were derived from a single case which inherently limits their generalizability and precludes comment on causality. The current findings should therefore be interpreted with caution. Nonetheless, by highlighting the potential clinical relevance of practice effects and the utility of very long delay intervals in the assessment of memory in ALF, we hope this case report will encourage further research involving larger studies (e.g., case series, group-based methodology).

### Recommendations

Future research is required to establish the utility of existing cognitive tests, commonly available to practicing neuropsychologists, for long-term forgetting. For example, tests like the BMIPB-II which already have a large normative dataset for healthy long-term forgetting could be a suitable comparison for future ALF clinical groups. With the availability of the original test data from the publishers then increasingly sophisticated approaches (e.g., propensity score matching) could be applied to closely match people with TLE with normative reference points.

In addition, further research is necessary to assess the rate of long-term forgetting (ALF) using commonly administered tests for visual memory, as most existing studies have focused primarily on verbal memory. Although some verbal memory tests include long-delay conditions to assess retention and detect ALF, visual memory tests generally lack these conditions. The absence of long-delay normative data for widely used visual memory assessments limits clinicians’ ability to evaluate ALF in the visual domain reliably (e.g., BMIPB-II does not have a visual memory long-delay condition).

### Conclusions

This case report highlights potential differences between long-delay memory tests and serial assessment practice effects to effectively detect ALF in individuals with TLE. Although both tests may provide added value over and above conventional memory assessment (20–40-min delay periods), a dissociation was observed on this occasion, in that only long-delay free recall memory tests showed an impairment. Assessing practice effects can provide additional value in that the presence of a memory trace (e.g., practice effects in the presence of poor long delay performance) can inform cognitive rehabilitation. Neuropsychologists are encouraged to consider incorporating this approach in evaluations of TLE patients.

## Supplementary Material

Supplementary_Figure_1_acaf077

## Data Availability

No data will be made available.
