# Supplementary figures and images for: Practice Effects and Long Delays: A Case Report Exploring a Novel Approach to Detecting Accelerated Long-Term Forgetting
Source: Arch Clin Neuropsychol. 2025 Aug 28;40(7):1444–52. doi: 10.1093/arclin/acaf077 (PMC12540254; doi:10.1093/arclin/acaf077)

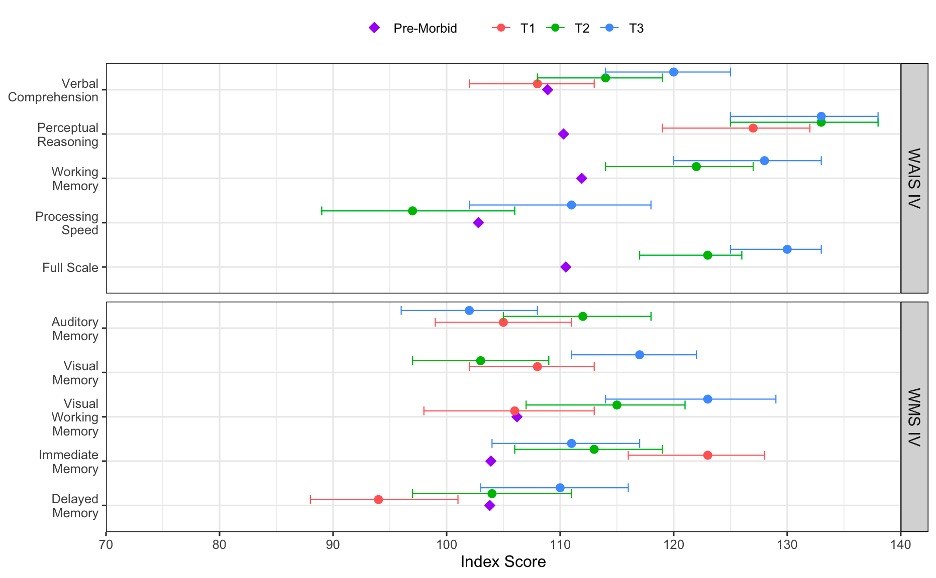

Supplement: Supplementary_Figure_1_acaf077 [file supplementary_figure_1_acaf077.jpeg]
